# Unique Reactivity of Transition Metal Atoms Embedded in Graphene to CO, NO, O_2_ and O Adsorption: A First-Principles Investigation

**DOI:** 10.3390/molecules201019540

**Published:** 2015-10-27

**Authors:** Minmin Chu, Xin Liu, Yanhui Sui, Jie Luo, Changgong Meng

**Affiliations:** School of Chemistry and State Key Laboratory of Fine Chemicals, Dalian University of Technology, Dalian 116024, China; E-Mails: molisa0618@mail.dlut.edu.cn (M.C.); zhuxuanlvluojie@mail.dlut.edu.cn (J.L.)

**Keywords:** graphene, defects, transition metal, single atom catalysis, CO oxidation

## Abstract

Taking the adsorption of CO, NO, O_2_ and O as probes, we investigated the electronic structure of transition metal atoms (TM, TM = Fe, Co, Ni, Cu and Zn) embedded in graphene by first-principles-based calculations. We showed that these TM atoms can be effectively stabilized on monovacancy defects on graphene by forming plausible interactions with the C atoms associated with dangling bonds. These interactions not only give rise to high energy barriers for the diffusion and aggregation of the embedded TM atoms to withstand the interference of reaction environments, but also shift the energy levels of TM-d states and regulate the reactivity of the embedded TM atoms. The adsorption of CO, NO, O_2_ and O correlates well with the weight averaged energy level of TM-d states, showing the crucial role of interfacial TM-C interactions on manipulating the reactivity of embedded TM atoms. These findings pave the way for the developments of effective monodispersed atomic TM composites with high stability and desired performance for gas sensing and catalytic applications.

## 1. Introduction

In graphene, the newest allotrope of carbon, all the C atoms are sp^2^ hybridized and interconnected in a two-dimensional honeycomb lattice through σ bonds, while the remaining C-p_z_ states that are not involved in the hybridization stand vertically to the lattices and conjugate strongly for formation of the delocalized π bonds. This special bonding feature among C atoms makes graphene a semimetal Dirac fermion system with a zero electronic band gap, good chemical stability, excellent electrical and thermal conductivity, as well as the remarkably high mechanical strength [1,2]. Owing to these unique properties, graphene has been extensively investigated for fabrication of electronic devices [3,4], supercapacitors [5,6], electrodes and sensors [7,8], *etc.*, as well as support material for dispersion of TM nanostructures for gas sensing and catalytic applications [9,10,11,12].

For TM composites for catalytic and gas sensing applications, high density of reaction sites with outstanding reactivity and stability are required to achieve superior performance [13]. One solution to this is to downsize the TM NPs to sub-nano scale or even single atoms to expose more unsaturated TM atoms that are the real reaction sites as determined by the delocalized nature of TM-d states [13]. The interfacial interaction may also promote the reactivity of the composites [14]. However, due to the delocalized π bonds, pristine graphene can only interact with ultrafine TM nanostructures through σ-π bonding and interaction of this type is normally below than −3 eV, which can hardly influence the electronic structure of TM [15]. Furthermore, the large surface energy is one of the key problems in fabrication of single TM atom or ultrafine TM structures, driven by which atomic diffusion and aggregation through Ostwald ripening may take place and result in formation of large TM structures with lowered performance and utilization of TMs. The energy barriers for TM atoms diffusion are typically lower than 1 eV which makes graphene hard to stabilize the deposited TM nanostructures [14,16,17]. In this sense, pristine graphene is not a good support material for fabrication of TM-graphene composites for gas sensing and catalytic applications.

The graphene materials used for chemical applications are generally made by oxidative exfoliation followed by reduction [18]. Driven by the large exothermicity for formation of CO and CO_2_, the exfoliation always generates various types of localized defects on the as synthesized graphene, whose evolution can be regulated with additional treatment such as electron beam radiation, chemical functionalization, *etc*. [19,20]. The existence and evolution of these defects provide a new platform to manipulate the reactivity of the deposited TM nanostructures through defect engineering [21,22,23,24]. The recent investigations on the electronic structure of sub-nanosized TM particles/reduced graphene oxide composites showed that these localized defect structures, such as monovacancies and multivacancies on graphene can not only act as strong trapping sites for TM nanostructures and inhibit their aggregation [25], but also determine the electronic structure as well as the reactivity of the composites through TM-graphene interfacial interaction [24,26]. As the size of TM nanostructures goes down, the impact of this interaction would in principle become more significant and this has been examplified by the recent successful invention of single TM atom catalysts for various reactions including CO oxidation [27,28,29]. Stabilization of TM atoms and sub-nanosized nanostructures has been realized experimentally [30,31,32], the electronic structure of TM atoms on graphene has been investigated before and predicted to be effective for hydrogen storage [33,34], while monodispersed Pt [27], Au [35], Cu [36] and Fe [37] atoms stabilized by vacancy defects over graphene have also been proposed to be efficient for CO oxidation. In this sense, it would be of great significance to investigate the electronic structure of these monodispersed TM atoms to generalize the role of interfacial TM-defect interaction in determining the reactivity of the formed composites to rationalize the design of single TM atom and subnanosized TM structures for gas sensing and catalytic applications.

In this work, we investigated the electronic structure of TM atoms (TM = Fe, Co, Ni, Cu and Zn) embedded in graphene and the adsorption of CO, NO, O_2_ and O on them by first-principles-based calculations. We showed that the plausible interactions between TM atoms and the defects on the graphene significantly enhance the binding of TM atoms and exclude the possibility for embedded TM atoms to aggregate and form large particles. These interactions also shift the energy levels of TM-d states and tune the reactivity of these atomic composites to small molecules. Further investigation shows that the adsorption of CO, NO, O_2_ and O correlates well the energy level of d states of the corresponding TM atoms. These findings pave the way for the developments of effective monodispersed atomic TM composites with high stability and desired performance for gas sensing and catalytic applications. The rest of the paper is organized as the following: the results are presented and discussed in Section 2, the theoretical methods and computational details are described in Section 3, and our conclusions are presented in Section 4.

## 2. Results and Discussion

We began with the atomic deposition of TM atoms on pristine graphene (PG) and the main results are summarized in Table 1. According to the hexagonal symmetry of the PG lattice, TM atoms can be deposited on the top of a C atom (Atop), on the middle of two adjacent C atoms (Brg) and above the center of the C_6_ ring (Hol). The calculated E_b_ for deposition of TM atoms on PG are in the range from 0.02 to 1.51 eV [33]. According to the E_b_ and deposition configurations, chemical bonds are formed upon atomic deposition of Fe, Co and Ni on PG, while Cu and Zn only bind PG through van der Waals interaction. We reinvestigated these cases using GGA-PBE functional with Grimme scheme for DFT-D correction and found that the contribution of van der Waals interaction is within 0.3 eV and will not alter their relative stability [33]. We also calculated the diffusion barriers of deposited TM atoms and found that the barriers on PG fall in the range of 0.01 to 0.44 eV, which indicates that the deposited TM atoms should be highly mobile even at moderate temperatures [16,38]. The fast diffusion of TM atoms makes PG less eligible for fabrication of atomic TM/graphene composites for sensing and catalytic applications.

**Table 1:** The most plausible structures, E_ad_ and diffusion barriers of TM atoms deposition on PG.

TM	E_b_ (eV) ^a^	E_a_ (eV) ^b^	*d_TM-C_* (Å) ^c^	*h* (Å) ^d^
Fe	−0.92 (Hol)	0.42	2.12	1.56
Co	−1.44 (Hol)	0.44	2.10	1.53
Ni	−1.51 (Hol)	0.21	2.12	1.56
Cu	−0.23 (Atop)	0.03	2.20	2.04
Zn	−0.02 (Hol)	0.01	3.02	2.78

^a^ The binding energy of TM atoms onto PG, calculated from Equation (1); ^b^ The barrier for diffusion of deposited TM atoms; ^c^ The nearest TM-C distance in the most plausible adsorption configuration as indicated; ^d^ The distance from TM to the PG plane.

**Figure 1:**
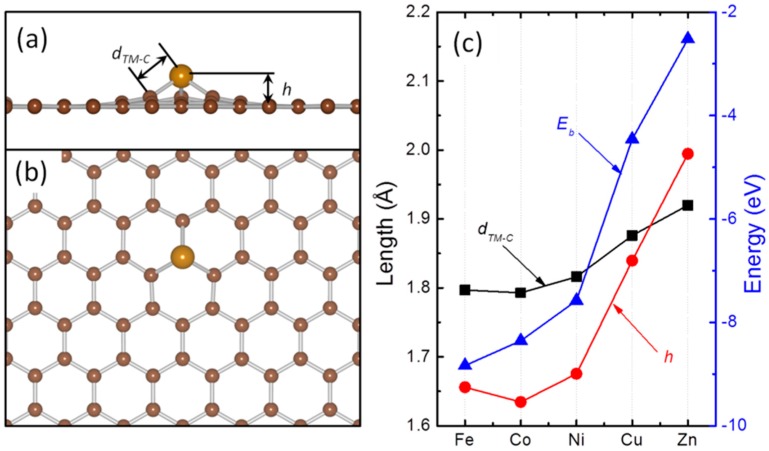
The side view (**a**) and top view (**b**) of the optimized structures of a TM atom embedded in graphene, and the evolution of the corrugation (*h*); the nearest TM-C distance (*d_TM-C_*) and binding energy (E_b_) when TM varies in Fe, Co, Ni, Cu and Zn (**c**).

Stabilization of these single TM atoms is one of the outstanding problems in fabrication of single TM atoms for chemical sensing and catalysis, as their ultrahigh surface energy will drive them to aggregate on the surface of the substrate resulting in formation of large TM NPs and low utilization of TMs [14]. One possible solution to this is to enhance the stability of the atomic TM-GN composites by strengthening the interaction at the TM-graphene interface that rises the diffusion barriers and tunes the atomic diffusion endothermic [25]. The graphene materials used for chemical applications are generally made through Hummer’s method. The exfoliation always generates various types of localized defects on the as synthesized graphene [19]. The existence and evolution of these defects provides a new platform to manipulate the reactivity of the deposited TM nanostructures. We previously studied the interaction of Ru atom with various defects observed on graphene sample synthesized through wet chemistry method and found that monovacancy (MG) is typical and these defects can effectively stabilize the monondispersed Ru atoms [22]. Inspired by previous findings, we then investigated the electronic structure of TM-MG composites with the structural and energetic information summarized in Figure 1.

Due to the large size of TM atoms as compared with C atom, the TM atoms will stand out of the basal plane of graphene and the embedment will cause strong corrugation within the graphene (Figure 1a,b). We characterized the stability of these composites with the E_b_ calculated as the difference between the TM atom in the substitution position and the energy of a reconstructed naked MG plus the energy of an isolated TM atom according to Equation (1) (Figure 1c). The TM-d states are screening the interaction between C-sp states and the TM states. Starting from Fe to Ni and with the increase of the population in TM-d states, the interaction C-sp states with TM states are weakened and so as the stability of the atomic composites, from −8.83 eV for Fe to −7.58 eV for Ni. As for Cu and Zn that have a closed d-shell, the involvement of TM-d states in TM-C bonding is quite limited due to the poor compatibility between these states and defect states of MG in energy space. The calculated E_b_ are −4.46 and −2.52 eV for Cu and Zn, respectively and are significantly unstable than those TM with a partially occupied d-shell. Resulting from these, the out-of-plane displacement (*h*, Figure 1a,c) is increasing from 1.63 (Fe) to 1.99 Å (Zn). Following this, the nearest TM-C distance (*d_TM-C_*, Figure 1a,c) also changes from 1.79 Å for Co to 1.92 Å for Zn. The small *d_TM-C_* and *h* in these TM-MG composites provide direct evidence for the formation of chemical bonding between the TM states and the dangling bonds localized on C atoms around the vacancy. In Figure 1c, the small *d_TM-C_* at Co can be attributed to the highly spin polarized nature of Co-d states due to the limited coordination in Co-MG [39].

**Figure 2:**
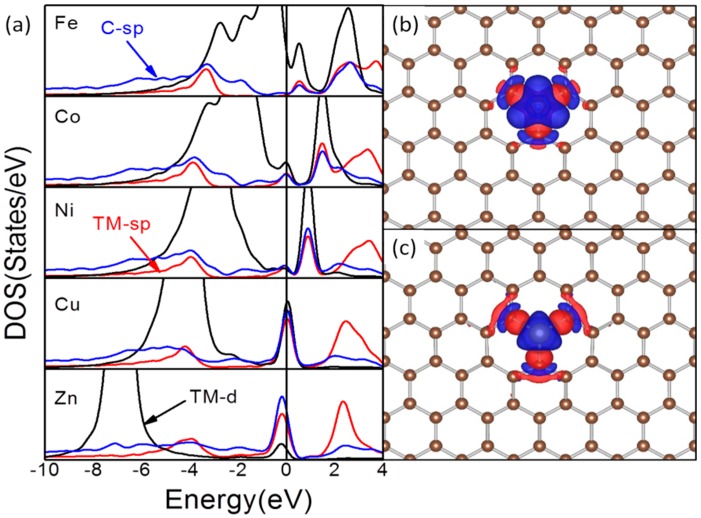
Density of states curves for TM-MG composites (**a**) and contour plots of differential charge density for Fe-MG (**b**) and Zn-MG (**c**) composites. In (**a**), the DOS curves of TM-d, TM-sp and C-sp states are in black, red and blue, respectively. The DOS curves are aligned with the calculated Fermi level (E_F_) which is set to zero. In (**b**,**c**), the contour value is ±3 × 10^−7^ a.u. The charge accumulation regions are in red and the charge depletion regions are in blue.

Comparing with TM atomic deposition on PG, the binding energies are significantly enhanced. The E_b_ for Fe-MG is −8.83 eV and is enhanced by about 9 times as compared with that on PG (−0.92 eV). Even for Cu, the E_b_ of Cu-MG is −4.46 eV and is enhanced by more than 10 times than that over the PG (−0.23 eV). The significant enhanced E_b_ on MG makes the diffusion of TM atoms endothermic. After embedment, Zn diffusion from the defect site becomes endothermic by ~2.50 eV, implying that the barrier for Zn atomic diffusion would be at least 2.50 eV. The endothermicity for diffusion of embedded Fe atoms is even enhanced to 7.91 eV. The significant enlarged diffusion barriers suggest that the atomic diffusion of embedded TM atoms cannot take place in conventional condition and thus ensure the chemical stability of these TM-MG composites for sensing and catalytic applications. The density of states (DOS) analysis was performed to understand the interfacial TM-C interactions, as shown in Figure 2a.

The DOS of MG is characterized by the sharp spikes around the E_F_ which are associated with the dangling bonds localized on the C atoms around the defect site [22]. After the embedment of TM atoms, the DOS peaks of these dangling bonds and valence states of TMs are significantly downshifted, get broadened and strong resonance among these states is obvious. This is a sign that these TM atoms are using their valence states to interact with the dangling bonds of MG. The degree of hybridization among TM-sp, TM-d and C-sp states shows a strong dependence on the population in TM-d states. This hybridization gives rise to the shoulder on the DOS curves of those TMs with a partially occupied d-shell at −4.0 eV where TM-d, TM-sp and C-sp have contributions. When the d-shell is fully occupied, the TM-d and TM-sp states are no longer compatible as shown by the large difference in energy levels of these states and the narrow distribution of TM-d state, and the interfacial interaction is mainly contributed by the TM-sp and C-sp. These hold for the relatively weak stability of embedded Cu and Zn atoms. We also plotted the differential charge density to visualize the charge transfer (Figure 2b,c). There are large charge accumulation regions at the TM-C interface while the charge depletion regions are on the TM and C atoms, showing that these TM-C interactions are of partially covalent nature. The shape of those charge depletion regions on both TM and C atoms confirms that the interfacial interaction is the contribution of TM valence states and the dangling bonds and is in line with the DOS analysis.

Due to the joint effect of interfacial interaction and the characteristics of the embedded TM atoms, the energy distribution of TM-d states varies with the TM. There is also a clear trend that with the increase of the population, the main peaks of TM-d states are shifting to lower energy. For all the TMs, the TM-d states are split into two parts due to the TM-MG interaction, one part is around the E_F_ and is partially occupied or unoccupied, while another part is occupied. The split is the smallest for Fe with a partially occupied d-shell and largest for Zn with a filled d-shell. It should be noted that for some cases, such as Cu and Fe, the TM-C interaction also shifts some of the TM-d states to the E_F_ [27,37]. The energy levels of TM-d states are known vital for the TM-adsorbate bonding and account for the superior reactivity of TM atoms at the edge and corners of TM nanostructures. The different distribution of TM-d states implies that these TM-MG composites would exhibit distinct reactivity to the adsorbates as compared with the bulk TMs.

We then used O_2_, O, CO and NO as adsorbates to probe the reactivity of these TM-MG composites. These adsorbates are also important reaction species for some widely investigated reactions including CO oxidation, oxygen reduction and conversion of nitrogen oxides [40]. The calculated O-O, C-O and N-O distances in freestanding O_2_, CO and NO are 1.23 Å, 1.14 Å, and 1.16 Å, respectively. On PG, O_2_ lying parallel to the graphene surface with the O-O axis parallel to the axis of two opposite C atoms in the same C_6_ ring is the most plausible. The calculated E_ad_ is less than −0.10 eV and the O-O bond length almost remains the same as the free O_2_ molecule. The stacking between the π states of O_2_ and PG accounts for this weak interaction. Due to the dominant role of π-π stacking in stabilization of CO and NO on PG, the most plausible adsorption configuration of CO and NO on PG are similar to that of O_2_. The calculated adsorption energies are less than 0.10 eV and the nearest distances between CO, NO and PG are all above 3.20 Å [41,42]. As for the adsorption of O on PG, the O atom lying above the middle of two adjacent C atoms is plausible and the calculated E_ad_ is −2.07 eV [42,43,44].

**Figure 3:**
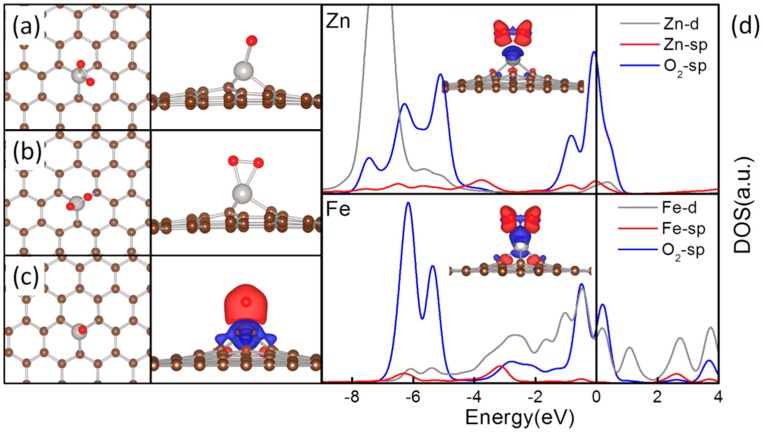
The top view and side view of Octa (**a**) and Tetr (**b**) configurations for O_2_ adsorption on TM-MGs, the top view of the configuration for O adsorption on Fe-MG and the corresponding contour plot of differential charge density (**c**); and the DOS curves of O_2_ adsorption on Fe-MG and Zn-MG (**d**) in Octa. In structures, the C atoms are in brown, the TM atoms are in silver and the O atoms are in red. The contour plots of differential charge density for O_2_ adsorption are shown as insets in (**d**). The charge accumulation regions are in red and the charge depletion regions are in blue. In (**d**), the DOS curves were aligned by the calculated E_F_ that was set to 0.

The most plausible adsorption configurations for O_2_ and O adsorption on TM-MGs are listed in Table 2. As the interfacial interaction split the TM-d states, the O_2_ can use its antibonding π states to interact with either the in-plane TM-d states of *t*_2g_ symmetry in a distorted octahedral (Octa) or to interact with the TM-d states of *t*_2_ symmetry in a tetrahedral (Tetr). This results in formation of two different types of adsorption configurations, as shown in Figure 3. O_2_ lies parallel to the substrate plane and two O-TM bonds are formed in-plane with and in the reverse direction of two C-TM bonds in Octa, while two O-TM bonds are formed in the plane vertical the substrate plane with the O_2_ standing immediately on top of the TM atom in Tetr. Due to the TM-C interactions and different hybridization between the TM-sp and TM-d states originated from the population in TM-d states and the TM-C interfacial interactions, the O_2_ adsorption is plausible in Octa for Co-MG, Ni-MG, Cu-MG and Zn-MG, and in Tetr for Fe-MG. As for O atom, it prefers to act as a ligand to interact directly with the TM atoms in a tetrahedral coordination environment.

**Table 2:** The structural and energetic parameters for O_2_ and O adsorption on TM-MG composites.

TM	O_2_					O		
	E_ad_^a^ (eV)	d_O-TM_^b^ (Å)	d_O-O_^c^ (Å)	d_C-TM_^d^ (Å)	C.E. ^e^	E_ad_^a^ (eV)	d_O-TM_^b^ (Å)	d_C-TM_^d^ (Å)
Fe	−2.05	1.85	1.39	1.82	Tetr	−2.09	1.62	1.82
Co	−1.74	1.90	1.37	1.80	Octa	−1.80	1.64	1.80
Ni	−1.52	1.94	1.37	1.84	Octa	−1.66	1.66	1.82
Cu	−1.30	1.92	1.36	1.89	Octa	−0.94	1.72	1.92
Zn	−0.71	2.02	1.35	1.97	Octa	−0.23	1.79	2.00

^a^ The adsorption energy calculated from Equation (2), the E_ad_ of O is calculated with respect to O_2_; ^b^ The minimum TM-O distance in the most plausible adsorption configuration of either O_2_ or O; ^c^ The O-O distance in the most plausible adsorption configuration of O_2_; ^d^ The minimum TM-C distance in the most plausible adsorption configuration of either O_2_ or O; ^e^ The notation of the most plausible adsorption configuration.

**Figure 4:**
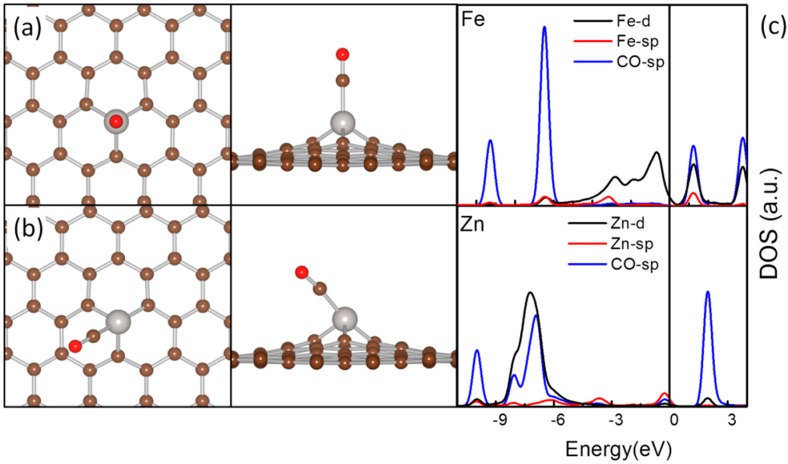
The top view (left panel) and side view (middle panel) of the Tetr’ (**a**) and Octa’ (**b**) configurations for CO and NO adsorption on TM-MGs, and the DOS curves for CO adsorption on Fe-MG and Zn-MG (**c**). In (**a**,**b**), the C atoms are in brown, the TM atoms are in silver and the O atoms are in red. In (**c**), the DOS curves were aligned by the calculated E_F_.

The most plausible adsorption configurations of NO and CO are listed in Table 3 with the typical adsorption structures shown in Figure 4a,b. They prefer to interact with the TM atoms with both their filled σ states associated with the lone pair on C or N for formation of σ-donation and their unfilled π states of antibonding character to accommodate the back-donated TM electrons for formation of the π-back donation (Figure 4c). These two interactions account for the stabilization of NO and CO on the embedded TMs. However, due to the Jahn-Teller distortion originated from the population in TM-d states, ligands of this type can interact with either axial components with *e_g_* symmetry of the TM-d states in a distorted octahedral with CO or NO along the direction reverse to a TM-C bond (Octa’, Figure 4b) or with the TM-d states with *t*_2_ symmetry in a distorted tetrahedral with adsorbates in the axial position (Tetr’, Figure 4a). The stability preference for CO and NO in these 2 configurations are also dependent on the population in TM-d states and their hybridization with the TM-sp states and C-sp state of MG which is regulated by the TM-C interfacial interaction. As the Jahn-Teller distortion is significant for TMs with a more than half-occupied TM-d states, the adsorbed CO is stable in Octa’ for Co, Ni and Cu. Different from CO, NO has an unpaired electron and the electron pairing upon adsorption will introduce additional stability and distortion to the coordination around the embedded TM atom. NO adsorption over Fe-MG and Ni-MG is plausible in the Octa’, while the Tetr’ is preferred for other TM-MGs.

**Table 3:** The structural and energetic parameters for CO and NO adsorption on TM-MG composites.

TM	CO			NO		
	E_ad_^a^ (eV)	d_C-TM_^b^ (Å)	d_C-O_^c^ (Å)	E_ad_^a^ (eV)	d_N-TM_^d^ (Å)	d_N-O_^e^ (Å)
Fe	−1.23	1.91	1.16	−1.30	1.77	1.19
Co	−1.10	1.89	1.16	−1.18	1.77	1.19
Ni	−1.12	1.87	1.16	−1.13	1.74	1.19
Cu	−1.13	1.87	1.16	−1.00	1.79	1.18
Zn	−0.94	1.94	1.16	−0.69	1.99	1.19

^a^ The adsorption energy calculated from Equation (2); ^b^ The minimum TM-C distance in the most plausible adsorption configuration of CO; ^c^ The C-O distance in the most plausible adsorption configuration of CO; ^d^ The minimum TM-N distance in the most plausible adsorption configuration of NO; ^e^ The N-O distance in the most plausible adsorption configuration of NO.

According to Table 2 and Table 3, it is apparent that the E_ad_ of all these adsorbates are dramatically enhanced as compared with their adsorption over PG. Furthermore, there are significant elongation of the O-O, C-O and N-O bonds in the plausible adsorption configurations, proving both the direction of the charge transfer is from the TM-MGs to the adsorbs and the activation of the these adsorbates. The variations of the TM-C distance after adsorption are within 0.05 Å, showing that due to the interfacial TM-C interactions, these TM-MG composites can survive in existence of these adsorbates.

**Figure 5:**
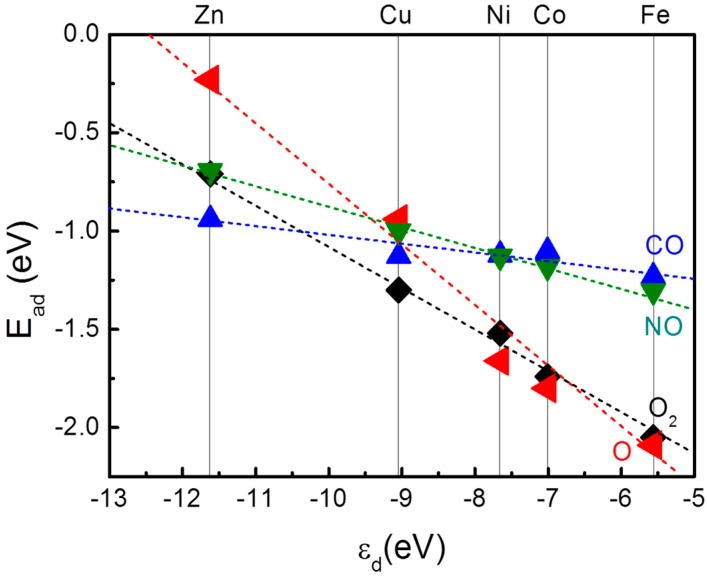
The calculated adsorption energy, E_ad_, plotted *vs.* calculated weight-average of TM-d states with respect to vacuum, ε_d_. The calculated E_ad_ correlates well with ε_d_. The dashed lines are to guide the eye.

We also noticed that for these adsorbates, there is a correlation between the E_ad_ of the adsorbates and the electronic structure of embedded TM. Adsorption of gaseous molecules and associated charge transfer are vital for various chemical processes including chemical sensing and catalysis. If we consider the adsorption of a gaseous molecule as a simple chemical reaction, according to the Frontier Molecular Orbital (FMO) theory proposed by Fuki *et al.*, the E_ad_ and the amount of charge transfered onto the TM composite would be determined by the energetic and spacial compatibility among the orbitals of the gas molecule and the TM-MG composite [45]. To simplify the discussion, we adapted the “d-band model” of proposed by Norskov and Hammer. In this model, the interaction of the adsorbate with the TM assembly is described as the joint effect of both the hybridization of adsorbate states with the TM-s states and the interaction with the TM-d states for production of states of either bonding or antibonding character. This theory has been successfully applied in extended TM systems ranging from bulk truncated surface of various TMs and TM alloys to even small TM clusters for reaction mechanism studies and catalyst design [46]. We aligned the energy levels of TM-d states of these TM-MG composites by the vacuum level and calculated the weight-averaged d-state centers (ε_d_). The adsorption energies of O_2_, O, CO and NO are plotted *vs.* the calculated ε_d_ in Figure 5, demonstrating a clear linear relationship. In this sense, the variation of E_ad_ of these adsorbates over TM-MG composites can be directly correlated with the ε_d_ determined by both the TM-C interfacial interactions and the population in the TM-d states.

## 3. Theoretical Methods

The first-principles based spin-polarized calculations were done using GGA-PBE functional and DSPP pseudopotentials with DND basis sets as implemented in the DMol^3^ package [39,47,48,49,50]. A hexagonal 9 × 9 supercell of pristine graphene was used to mimic the graphene and the TM-graphene composites. TM atoms were introduced by substituting C atoms. The minimum distance between the graphene sheet and its mirror images is set to be larger than 20 Å to avoid the interactions among the periodic images. We used empirical potential to preoptimize the substrate and the embedded composites and then had them fully relaxed using DMol^3^ until the residue forces were below 1 × 10^−2^ eV/Å [51,52]. A Γ centered 4 × 4 × 1 *k*-point grid was used for the Brillouin zone sampling during geometric optimization and a 20 × 20 × 1 *k*-point grid was used for electronic structure analysis [53]. All calculations were performed with a convergence criterion of 2 × 10^−4^ eV on the total energy and a real-space global orbital cutoff of 4.5 Å. The reaction barriers were calculated with the synchronous method with conjugated gradient refinements [54]. With the above setup, the minimum C-C distance in pristine graphene is 1.42 Å [1,55].

The binding energy (E_b_) of TM atom onto grapheme (GN) is calculated as the energy difference between the TM atomically deposited graphene (TMGN) and the separated graphene plus the freestanding TM atom, following Equation (1):

(1) $${\text{E}}_{\text{b}}{\text{ = E}}_{\text{TMGN}}\;-\;\left({\text{E}}_{\text{TM}}{\text{ + E}}_{\text{GN}}\right)$$

For the study concerning adsorption of CO, NO, O_2_, O, *etc.*, the adsorption energy (E_ad_) is calculated as the energy difference between the species absorbed TM deposited graphene and the gaseous species plus the bare TMGN, following Equation (2):

(2) $${\text{E}}_{\text{ad}}{\text{ = E}}_{\text{adsorbate + TMGN}}\;-\;\left({\text{E}}_{\text{adsorbate}}{\text{ + E}}_{\text{TMGN}}\right)$$

## 4. Conclusions

We addressed the electronic structure of transition metal (TM, TM = Fe, Co, Ni, Cu and Zn)-atoms embedded in graphene and the adsorption of CO, NO, O_2_ and O on them by first-principles-based calculations. Due to the plausible interaction between TM atoms and the defects on the graphene, the binding of TM atoms onto MG are significantly enhanced, which not only ensure the high stability of the embedding, but also dramatically rise the barrier for embedded TM atoms to diffuse and aggregate. This interaction also shifts the energy levels of TM-d states and regulates the reactivity of these TM-MG composites to small molecules. Further investigation shows that the adsorption of CO, NO, O_2_ and O correlates well the weight averaged energy level of TM-d states of the corresponding TM-MG. As the interfacial C-TM interactions play an important role in regulating the reactivity of the embedded TM atoms, any effect that involves the interactions with the TM atoms, such as doping or co-doping with N, B, P, S and other heteroelements, controlled fabrication of defects on the graphenic support, introduction of co-adsorbents, *etc.*, may help to optimize the reactivity of these embedded TM atoms to the adsorption of gaseous molecules. These findings pave the way for the development of monodispersed TM atomic composites for gas sensing and catalytic applications.

## References

[1] Castro Neto A.H., Guinea F., Peres N.M.R., Novoselov K.S., Geim A.K. (2009). The electronic properties of graphene. Rev. Mod. Phys..

[2] Novoselov K.S., Geim A.K., Morozov S.V., Jiang D., Zhang Y., Dubonos S.V., Grigorieva I.V., Firsov A.A. (2004). Electric field effect in atomically thin carbon films. Science.

[3] Schwierz F. (2010). Graphene transistors. Nat. Nanotechnol..

[4] Avouris P., Chen Z.H., Perebeinos V. (2007). Carbon-based electronics. Nat. Nanotechnol..

[5] Zhu Y.W., Murali S., Stoller M.D., Ganesh K.J., Cai W.W., Ferreira P.J., Pirkle A., Wallace R.M., Cychosz K.A., Thommes M. (2011). Carbon-based supercapacitors produced by activation of graphene. Science.

[6] Wang Y., Shi Z.Q., Huang Y., Ma Y.F., Wang C.Y., Chen M.M., Chen Y.S. (2009). Supercapacitor devices based on graphene materials. J. Phys. Chem. C.

[7] Shao Y.Y., Wang J., Wu H., Liu J., Aksay I.A., Lin Y.H. (2010). Graphene based electrochemical sensors and biosensors: A review. Electroanalysis.

[8] Huang X., Zeng Z.Y., Fan Z.X., Liu J.Q., Zhang H. (2012). Graphene-based electrodes. Adv. Mater..

[9] Huang X., Yin Z.Y., Wu S.X., Qi X.Y., He Q.Y., Zhang Q.C., Yan Q.Y., Boey F., Zhang H. (2011). Graphene-based materials: Synthesis, characterization, properties, and applications. Small.

[10] Julkapli N.M., Bagheri S. (2015). Graphene supported heterogeneous catalysts: An overview. Int. J. Hydrog. Energy.

[11] Cheng Y., Fan Y., Pei Y., Qiao M. (2015). Graphene-supported metal/metal oxide nanohybrids: Synthesis and applications in heterogeneous catalysis. Catal. Sci. Technol..

[12] Fan X.B., Zhang G.L., Zhang F.B. (2015). Multiple roles of graphene in heterogeneous catalysis. Chem. Soc. Rev..

[13] Yang F., Deng D.H., Pan X.L., Fu Q., Bao X.H. (2015). Understanding nano effects in catalysis. Natl. Sci. Rev..

[14] Yang X.F., Wang A.Q., Qiao B.T., Li J., Liu J.Y., Zhang T. (2013). Single-atom catalysts: A new frontier in heterogeneous catalysis. Acc. Chem. Res..

[15] Tang Q., Zhou Z., Chen Z.F. (2013). Graphene-related nanomaterials: Tuning properties by functionalization. Nanoscale.

[16] Chan K.T., Neaton J.B., Cohen M.L. (2008). First-principles study of metal adatom adsorption on graphene. Phys. Rev. B.

[17] Krasheninnikov A.V., Lehtinen P.O., Foster A.S., Pyykko P., Nieminen R.M. (2009). Embedding transition-metal atoms in graphene: Structure, bonding, and magnetism. Phys. Rev. Lett..

[18] Hummers W.S., Offeman R.E. (1958). Preparation of graphitic oxide. J. Am. Chem. Soc..

[19] Meyer J.C., Kisielowski C., Erni R., Rossell M.D., Crommie M.F., Zettl A. (2008). Direct imaging of lattice atoms and topological defects in graphene membranes. Nano Lett..

[20] Wang Z., Zhou Y.G., Bang J., Prange M.P., Zhang S.B., Gao F. (2012). Modification of defect structures in graphene by electron irradiation: *Ab initio* molecular dynamics simulations. J. Phys. Chem. C.

[21] Liu X., Yao K.X., Meng C.G., Han Y. (2012). Graphene substrate-mediated catalytic performance enhancement of Ru nanoparticles: A first-principles study. Dalton Trans..

[22] Liu X., Sui Y., Meng C., Han Y. (2014). Tuning the reactivity of Ru nanoparticles by defect engineering of the reduced graphene oxide support. RSC Adv..

[23] Liu X., Li L., Meng C., Han Y. (2012). Palladium Nanoparticles/defective graphene composites as oxygen reduction electrocatalysts: A first-principles study. J. Phys. Chem. C.

[24] Liu X., Meng C.G., Han Y. (2013). Defective graphene supported MPd12 (M = Fe, Co, Ni, Cu, Zn, Pd) nanoparticles as potential oxygen reduction electrocatalysts: A first-principles study. J. Phys. Chem. C.

[25] Liu X., Meng C.G., Han Y. (2012). Substrate-mediated enhanced activity of Ru nanoparticles in catalytic hydrogenation of benzene. Nanoscale.

[26] Liu X., Meng C., Han Y. (2012). Unique reactivity of Fe nanoparticles-defective graphene composites toward NHx (x = 0, 1, 2, 3) adsorption: A first-principles study. Phys. Chem. Chem. Phys..

[27] Liu X., Sui Y., Duan T., Meng C., Han Y. (2014). CO oxidation catalyzed by Pt-embedded graphene: A first-principles investigation. Phys. Chem. Chem. Phys..

[28] Yao K.X., Liu X., Li Z., Li C.C., Zeng H.C., Han Y. (2012). Preparation of Ru nanoparticles/defective graphene composite as a highly efficient arene hydrogenation catalyst. ChemCatChem.

[29] Liu X., Sui Y., Duan T., Meng C., Han Y. (2015). Monodispersed Pt atoms anchored on *N*-doped graphene as efficient catalysts for CO oxidation: A first-principles investigation. Catal. Sci. Technol..

[30] Qiao B., Wang A., Yang X., Allard L.F., Jiang Z., Cui Y., Liu J., Li J., Zhang T. (2011). Single-atom catalysis of CO oxidation using Pt1/FeOx. Nat. Chem..

[31] Wei H.S., Liu X.Y., Wang A.Q., Zhang L.L., Qiao B.T., Yang X.F., Huang Y.Q., Miao S., Liu J.Y., Zhang T. (2014). FeOx-supported platinum single-atom and pseudo-single-atom catalysts for chemoselective hydrogenation of functionalized nitroarenes. Nat. Commun..

[32] Guo X., Fang G., Li G., Ma H., Fan H., Yu L., Ma C., Wu X., Deng D., Wei M. (2014). Direct, nonoxidative conversion of methane to ethylene, aromatics, and hydrogen. Science.

[33] Manadé M., Viñes F., Illas F. (2015). Transition metal adatoms on graphene: A systematic density functional study. Carbon.

[34] Valencia H., Gil A., Frapper G. (2015). Trends in the hydrogen activation and storage by adsorbed 3d transition metal atoms onto graphene and nanotube surfaces: A DFT study and molecular orbital analysis. J. Phys. Chem. C.

[35] Lu Y.H., Zhou M., Zhang C., Feng Y.P. (2009). Metal-embedded graphene: A possible catalyst with high activity. J. Phys. Chem. C.

[36] Song E.H., Wen Z., Jiang Q. (2011). CO catalytic oxidation on copper-embedded graphene. J. Phys. Chem. C.

[37] Li Y.F., Zhou Z., Yu G.T., Chen W., Chen Z.F. (2010). CO catalytic oxidation on iron-embedded graphene: Computational quest for low-cost nanocatalysts. J. Phys. Chem. C.

[38] Sevincli H., Topsakal M., Durgun E., Ciraci S. (2008). Electronic and magnetic properties of 3d transition-metal atom adsorbed graphene and graphene nanoribbons. Phys. Rev. B.

[39] Valencia H., Gil A., Frapper G. (2010). Trends in the adsorption of 3d transition metal atoms onto graphene and nanotube surfaces: A DFT study and molecular orbital analysis. J. Phys. Chem. C.

[40] Liu X., Duan T., Meng C., Han Y. (2015). Pt atoms stabilized on hexagonal boron nitride as efficient single-atom catalysts for CO oxidation: A first-principles investigation. RSC Adv..

[41] Zhou M., Lu Y.H., Cai Y.Q., Zhang C., Feng Y.P. (2011). Adsorption of gas molecules on transition metal embedded graphene: A search for high-performance graphene-based catalysts and gas sensors. Nanotechnology.

[42] Leenaerts O., Partoens B., Peeters F.M. (2008). Adsorption of H_2_O, NH_3_, CO, NO_2_, and NO on graphene: A first-principles study. Phys. Rev. B.

[43] Zhang Y.-H., Chen Y.-B., Zhou K.-G., Liu C.-H., Zeng J., Zhang H.-L., Peng Y. (2009). Improving gas sensing properties of graphene by introducing dopants and defects: A first-principles study. Nanotechnology.

[44] Wu M., Liu E.Z., Jiang J.Z. (2008). Magnetic behavior of graphene absorbed with N, O, and F atoms: A first-principles study. Appl. Phys. Lett..

[45] Fukui K., Yonezawa T., Shingu H. (1952). A molecular orbital theory of reactivity in aromatic hydrocarbons. J. Chem. Phys..

[46] Hammer B., Norskov J.K. (1995). Electronic factors determining the reactivity of metal surfaces. Surf. Sci..

[47] Delley B. (1990). An all-electron numerical-method for solving the local density functional for polyatomic-molecules. J. Chem. Phys..

[48] Delley B. (2000). From molecules to solids with the DMol(3) approach. J. Chem. Phys..

[49] Perdew J.P., Burke K., Ernzerhof M. (1996). Generalized gradient approximation made simple. Phys. Rev. Lett..

[50] Delley B. (2002). Hardness conserving semilocal pseudopotentials. Phys. Rev. B.

[51] Liu X., Meng C.G., Liu C.H. (2006). Molecular dynamics study on superheating of Pd at high heating rates. Phase Transit..

[52] Liu X., Meng C.G., Liu C.H. (2004). Melting and superheating of Ag at high heating rate. Acta Phys. -Chim. Sin..

[53] Monkhorst H.J., Pack J.D. (1976). Special points for Brillouin-zone integrations. Phys. Rev. B.

[54] Govind N., Petersen M., Fitzgerald G., King-Smith D., Andzelm J. (2003). A generalized synchronous transit method for transition state location. Comput. Mater. Sci..

[55] Gajdos M., Eichler A., Hafner J. (2004). CO adsorption on close-packed transition and noble metal surfaces: Trends from ab initio calculations. J. Phys. -Condens. Matter.

